# Drug-Induced Liver Injury: Cascade of Events Leading to Cell Death, Apoptosis or Necrosis

**DOI:** 10.3390/ijms18051018

**Published:** 2017-05-09

**Authors:** Andrea Iorga, Lily Dara, Neil Kaplowitz

**Affiliations:** 1 Research Center for Liver Disease, Keck School of Medicine, University of Southern California, Los Angeles, CA 90033, USA; iorga@usc.edu (A.I.); dara@usc.edu (L.D.); 2 Division of Gastrointestinal and Liver Diseases, Department of Medicine, Keck School of Medicine, University of Southern California, Los Angeles, CA 90033, USA

**Keywords:** hepatotoxicity, human leukocyte antigen (HLA), adaptation, acetaminophen, DILI, apoptosis, necrosis

## Abstract

Drug-induced liver injury (DILI) can broadly be divided into predictable and dose dependent such as acetaminophen (APAP) and unpredictable or idiosyncratic DILI (IDILI). Liver injury from drug hepatotoxicity (whether idiosyncratic or predictable) results in hepatocyte cell death and inflammation. The cascade of events leading to DILI and the cell death subroutine (apoptosis or necrosis) of the cell depend largely on the culprit drug. Direct toxins to hepatocytes likely induce oxidative organelle stress (such as endoplasmic reticulum (ER) and mitochondrial stress) leading to necrosis or apoptosis, while cell death in idiosyncratic DILI (IDILI) is usually the result of engagement of the innate and adaptive immune system (likely apoptotic), involving death receptors (DR). Here, we review the hepatocyte cell death pathways both in direct hepatotoxicity such as in APAP DILI as well as in IDILI. We examine the known signaling pathways in APAP toxicity, a model of necrotic liver cell death. We also explore what is known about the genetic basis of IDILI and the molecular pathways leading to immune activation and how these events can trigger hepatotoxicity and cell death.

## 1. Introduction

Drug-induced liver injury (DILI) is a frequent cause of liver injury, which presents with a broad spectrum of manifestations [1,2], the most serious of which is hepatocellular death leading to acute liver failure (ALF) following drug intake [3,4,5]. DILI has been linked to over 1000 drugs, and is one of the most frequently cited reason for drug non-approval, withdrawal, abandonment and post-marketing regulatory actions [6,7,8]. DILI can present clinically with multiple manifestations such as acute hepatitis, cholestasis and jaundice, nodular regenerative hyperplasia, or sinusoidal obstruction syndrome, although almost any clinical pathological acute or chronic patterns of liver disease may occur [1,2]. DILI can also present as a silent subclinical disease, detected during routine blood tests [3]. Gender and age are determinants of pattern and severity of injury; female gender is a risk factor for developing hepatocellular DILI and acute liver failure while age greater than 60 predisposes to the development of cholestastatic DILI with a more indolent course [9]. Agents which induce liver toxicity and DILI can be categorized as either intrinsic liver toxins with dose-dependent and predictable adverse effects or idiosyncratic [10]. Idiosyncratic Drug-Induced Liver Injury (IDILI) is by definition an unpredictable injury due to a drug, usually occurring after a relatively long latency and only in a small proportion of exposed individuals. IDILI is not strictly dose dependent despite exhibiting a dose threshold of 50–100 mg [11,12]. IDILI is a significant health problem due to its unpredictability, potential cause for mortality, and poorly understood pathogenesis [13].

The ensuing liver injury from drug hepatotoxicity (whether idiosyncratic or predictable) results in hepatocyte cell death and inflammation. The cascade of events leading to cell demise depends on the cell death subroutine activated. In the liver, the dominant forms of hepatocyte cell death are apoptosis and necrosis. The mechanism of cell death leading to hepatocytes demise in direct DILI is likely due to the induction of organelle stress (such as endoplasmic reticulum (ER) and mitochondrial stress) leading to necrosis or apoptosis, while cell death in IDILI is usually the result of engagement of the innate and adaptive immune system (likely apoptotic), involving death receptors (DR). While the full mechanisms of cell death from drug hepatotoxicity have not been fully elucidated, we will review what is known in the field and examine the general principles and players in these events which have been described in other contexts or liver disease models.

## 2. Cell Death in Drug-Induced Liver Injury (DILI)

**Figure 1:**
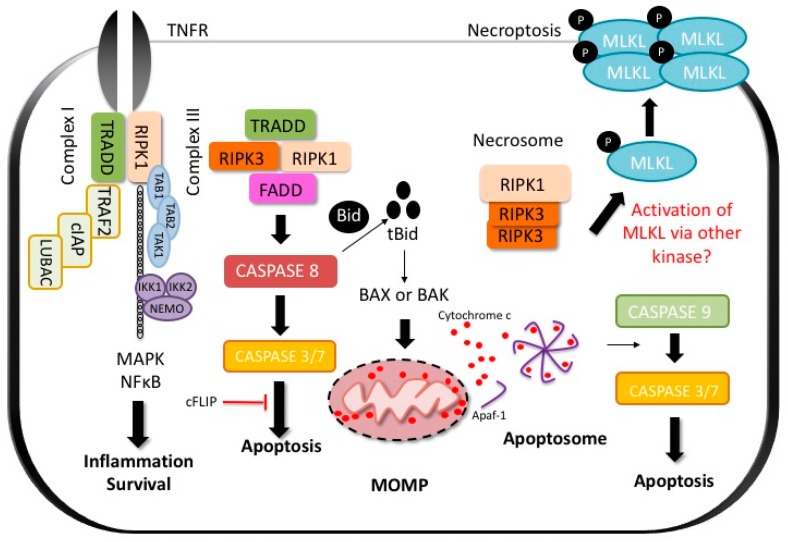
Tumor necrosis factor (TNF) receptor-mediated cell death. TNF binding to TNF receptor (TNFR) results in the formation of complex I which includes TNFR-associated death domain (TRADD), receptor interacting protein kinase-1 (RIPK1), TNFR-associated factor 2 (TRAF2), cellular inhibitor of apoptosis 1 (cIAP1), cellular inhibitor of apoptosis 2 (cIAP2) and linear ubiquitin chain assembly complex (LUBAC). RIPK1 is ubiquitinated resulting in the formation of a platform and recruitment of the IκB kinase (IKK) complex (NFκB Essential Modulator (NEMO), IKK1 and IKK2) and TAB (TAK1-binding protein)/TAK-1 (transforming growth factor-β-activated kinase 1) complexes. This results in the activation of NFκB and mitogen-activated protein kinase (MAPK). For apoptosis to occur, the cytoplasmic Complex II is formed which can have two forms. Complex IIa is made up of TRADD, FADD (fas-associated protein with death domain) and caspase-8 and complex IIb consists of RIPK1/RIPK3, FADD, caspase 8 and FLICE-like inhibitory protein long form (FLIPL). Complex II leads to caspase-8-mediated activation of caspase 3/7 and apoptosis in type I cells such as lymphocytes. In type II cells such as hepatocytes, caspase 8 mediates cleavage of Bid to cleaved Bid (tBid) resulting in Bax- (Bcl-2-like protein 4) and BAK-mediated (Bcl-2 homologous antagonist/killer) mitochondrial outer membrane permeabilization (MOMP) and Cytochrome C release from mitochondria. Cytochrome C binds to apoptotic peptidase activating factor-1 (APAF-1) releasing its auto-inhibitory hold. APAF-1 oligomerizes to form a wheel-like structure called the apoptosome which activates caspase 9. Caspase 9 in turn activates the effector caspases (caspase 3/7) to induce apoptosis. In certain cells, when caspase-8 is inhibited, the cell switches to an alternative form of cell death termed necroptosis. For necroptosis to occur, RIPK1, RIPK3 and mixed lineage domain like (MLKL) form the necrosome. RIPK3 phospho-activates MLKL, leading to its oligomerization and translocation to the cell membrane to induce pore opening and oncolysis. cFLIP: cellular FLICE-like inhibitory protein.

Hepatotoxicity results in liver cell death, which can be apoptotic or necrotic depending on the signaling pathways activated (Figure 1). The mechanisms of cell death and the subroutine involved largely depend on the drug or toxin, the magnitude of liver injury, and the initiating mechanism of cell death. In hepatocytes, direct toxicity and intracellular organelle stress (ER stress or mitochondrial toxicity) can activate the intrinsic pathway of apoptosis via mitochondrial outer membrane permeabilization (MOMP) or lead to necrosis by mitochondrial permeability transition (MPT). With extensive liver damage, it can be difficult to distinguish the predominant form of cell death in histology [14]. While apoptosis involves caspases, chromatin fragmentation and the phagocytosis of cell bodies which minimizes inflammation, necrosis is defined by cell swelling and the rupture of the plasma membrane (oncosis) which promotes an inflammatory response. The loss of hepatocytes is the main event contributing to the liver injury, but bile duct epithelial cells [15] or sinusoidal cells [16,17] may also be targeted.

### 2.1. Apoptosis

There are two routes to apoptosis: the extrinsic (death receptor initiated) and the intrinsic (mitochondrial) pathways. In both instances, the activation of executioner caspases (caspase 3 and 7) results in proteolysis, pyknosis (chromatin condensation) and karyorrhexis (nuclear fragmentation), which are the classic morphologic features of apoptosis [18]. The extrinsic pathway of apoptosis is activated when DR such as tumor necrosis factor receptor (TNFR), FAS (cluster of differentiation 95 (CD95)), TNF-related apoptosis-inducing ligand receptor (TRAIL-R1 and R2), are engaged by their ligands resulting in caspase 8 activation. All members of the death receptor family are highly expressed in the liver. Activation of CD8^+^ (cluster of differentiation 8) cytotoxic T lymphocytes and cytokines (free and membrane-bound) results in immune-mediated killing of hepatocytes. These T cells which are major histocompatibility complex (MHC) class I–restricted are largely responsible for hepatocyte apoptosis. However, other immune cells such as natural killer (NK) cells, natural killer T cells (NKT) and Kupffer cells (KC) also contribute [19,20,21]. When DR are activated by their respective ligands, they oligomerize and recruit proteins to their cytoplasmic domains (Figure 1).

As an example, we will discuss the pathways activated upon TNFR stimulation by its ligands as it is among the most extensively studied signaling cascades in molecular biology (Figure 1). When TNF binds its receptor, multiple proteins are recruited to a membrane-bound supramolecular structure termed complex 1 or death inducing complex (DISC) [22]. These include TNFR-associated death domain (TRADD), receptor interacting protein kinase-1 (RIPK1), cellular inhibitor of apoptosis 1 and 2 (cIAP1 and 2), TNFR-associated factor 2 (TRAF2) or TRAF5 [4,23]. cIAPs, which are E3 ubiquitin ligases, catalyse the Lys63-linked polyubiquitination of RIPK1 which serves as a scaffold for NFκB activation through transforming growth factor-β-activated kinase 1 (TAK1) and TAK1-binding protein 2 and 3 (TAB2 and TAB3) [23,24]. NFκB activation leads to inflammation and cell survival [24]. For death induction, a second complex, receptor-free complex II, consists of FADD, RIPK1 or TRADD, and caspase-8 assembles in the cytoplasm [24]. Activation of the caspase-8, an initiator caspase, leads to the activation of the executioner caspases (caspase-3 and 7) [23,24]. In many cells such as hepatocytes, formation of complex II is constitutively blocked by Fas-associating protein with death domain-like interleukin-1 β-converting enzyme (FLICE) inhibitory protein (c-FLIP) and NFκB target genes such as *A20* [24,25]. The robust NFκB activation in these cells renders them highly resistant to the lethal actions of TNF. However, this resistance can be overcome by translation or transcription inhibitors (such as actinomycin-D or galactosamine), generation of free radicals and reactive oxygen species (ROS), as well as glutathione (GSH) depletion [26,27,28,29].

In type I cells such as lymphocytes, activation of caspase-8 is sufficient for the activation of caspase-3 and 7 resulting in apoptosis. However, in type 2 cells such as hepatocytes, the induction of the extrinsic pathway of apoptosis requires mitochondrial participation and caspase-8-mediated cleavage of Bid, a Bcl2 protein. Cleaved Bid (tBid) and Bim activate proapoptotic Bcl2 family members Bax and Bak leading to MOMP and release of intermembrane proteins such as cytochrome c [23]. The release of cytochrome c activates the apoptosome by releasing apoptotic peptidase activating factor-1 (APAF-1) from its auto-inhibitory conformation [30,31]. APAF-1 then forms a wheel-like structure called the apoptosome which promotes self-activation of caspase 9, which in turn cleaves executioner caspases resulting in apoptosis (Figure 1) [32,33].

### 2.2. Regulated Necrosis and Necroptosis

Necrosis of hepatocytes, to a large part, also involves activation of cellular signaling pathways. However, the release of intracellular components during necrotic cell lysis causes ion imbalance, mitochondrial dysfunction, adenosine triphosphate (ATP) depletion and elicits an inflammatory response. While drug-induced hepatocellular necrosis is currently being studied, the mechanisms are still not well understood [14]. Necrotic cell death was considered incidental and a form of non-regulated cell death until increasing evidence demonstrated that necrosis can be tightly regulated and pharmacologic inhibition or genetic manipulation can interfere with the death process [3].

Necroptosis is a specific form of regulated necrosis initiated by TNF super family member receptor activation in the presence of caspase inhibitors such as Z-VAD-FMK and mediated through the activation of the pseudokinase mixed lineage domain such as (MLKL) by RIPK1 and RIPK3 interaction [34,35,36,37,38]. Necroptosis requires the kinase activity of RIPK1 and is inhibited by the necrostatins (nec) which are RIPK1 kinase inhibitors [35,39]. RIPK1 recruits RIPK3, through the interaction of their RIP homology interaction motif (RHIM). RIPK3 then activates MLKL by phosphorylation and p-MLKL subsequently translocates to the cell membrane where it oligomerizes and inserts itself executing necroptosis through breaching of the cell membrane (Figure 1) [36,38]. RIPK3 is the only known activator of MLKL, although recently MLKL activation was observed independent of RIPK3 in the Con A model of inflammatory hepatocyte death [40]. The kinase responsible for MLKL activation was not identified (more on this below). Given that hepatocytes do express MLKL in the absence of RIPK3, there remains the possibility that a non-canonical pathway to necroptosis activation exists in these cells. Despite reports citing increased expression of necroptosis proteins in liver biopsy specimens from patients with liver disease [38,41], the role of necroptosis (canonical and non-canonical) in human liver disease and DILI remains largely unknown [42].

Not all cell types can undergo necroptosis; the presence of RIPK3 dictates a cell’s ability to undergo programmed necrosis, and cells lacking RIPK3 such as HeLa and Hek293 are unable to activate necroptosis [43,44]. Interestingly, normal hepatocytes do not express RIPK3 [40,45,46,47]. Since APAP DILI results in a morphologically necrotic form of liver cell death, the murine APAP model has been used to study the role of necroptosis in the liver [42]. Whether necroptosis participates in APAP DILI has been controversial since nec-1 has been reported to prevent APAP toxicity [47,48,49,50], while MLKL and RIPK3 knockout did not prevent cell death in APAP DILI [40,42,47]. Knockdown of RIPK1 protects against APAP DILI and hepatocyte necrosis in vivo, both in wild type and RIPK3^−/−^ mice [47]. Therefore, while RIPK1 may participate in cell death pathways during APAP toxicity, necroptosis is not activated by APAP [42,47]. Thus, the role of RIPK1 in APAP toxicity seems to be independent of its role in necroptosis. RIPK1 is a key kinase with multiple biological functions, such as participation in canonical NFκB and MAPK activation as well as inflammatory responses [34]. The exact mechanism of its participation in APAP toxicity needs to be elucidated but seems to be upstream of c-Jun terminal kinase (JNK) activation [47].

### 2.3. Autophagy

Autophagic cell death is a controversial topic that has also been investigated in DILI. Autophagy is the lysosomal degradation pathway through which cell content including damaged organelles and protein is recycled. Autophagy is executed through the formation of double membrane vesicles (autophagosomes) originating from endoplasmic reticulum or the cell membrane, that fuse with and deliver their cytoplasmic cargo to lysosomes for degradation by lysosomal hydrolases. Autophagy plays an important role in programmed cell death, especially in close collaboration with apoptosis. However, autophagy is not solely a cell death mode. It is also a pivotal process in development, immune defense, tumor suppression and many other cellular processes [51]. In fact, it is mainly a cell survival mechanism enabling the cell to recycle its contents. Knockout of *ATG* genes (responsible for autophagy) in most instances is associated with acceleration of cell death rather than its inhibition or prevention [51]. The role of autophagy has been studied in APAP-induced hepatotoxicity [52]. Since APAP toxicity primarily targets and damages mitochondria, which generate free radicals and ROS, mitophagy (a form of autophagy selectively involving mitochondria) was shown to ameliorate APAP DILI [52]. APAP resulted in the formation of autophagosomes that engulfed mitochondria and pharmacological inhibition of autophagy by 3-methyladenine or chloroquine exacerbated APAP toxicity. This effect was attenuated by rapamycin, even if administered two hours after APAP [52].

### 2.4. Other Forms of Cell Death: (Pyroptosis and Ferroptosis)

Recently, other subroutines of cell death such as pyroptosis and ferroptosis have been described but not much is known about these entities in liver disease. Pyroptosis is a regulated and inflammatory form of cell death, first named in 2001 for the Greek words “pyro” (fire or fever) and “ptosis” (to fall). It has evolved as a way of removing invading pathogens from the body but excessive activation can result in inflammatory cell death. Pyroptosis is dependent on the activation of the inflammatory caspases 1, 4, 5, and 12 in humans (caspase-11 in mice), which are activated through intracellular lipopolysaccharide (LPS) or the inflammasome (caspase-1). These inflammatory caspases then cleave the N-terminus of Gasdermin D (GSDMD), releasing it from the autoinhibitory C domain. Activated GSDMD-N then translocates and binds to lipids in the plasma membrane forming pores, leading to cell death [53,54,55]. This has been viewed as a way of releasing cytokines from macrophages in the context of infection. Little is known about this mechanism in hepatocytes.

Ferroptosis is an oxidative, iron-dependent form of cell death triggered by inactivation of cellular glutathione (GSH)-dependent antioxidant defenses such as glutathione precursor transport, GSH synthesis and GSH peroxidase 4 (GPX4). GPX4 is a membrane lipid peroxidase and largely prevents lipid peroxidation. Deletion of GPX4 results in ferroptotic cell death that can be rescued by ferrostatins (Fer-1) and iron chelators, confirming the role of GPX4 in this cell death subroutine [56,57,58]. Ferroptosis has been suggested to occur in APAP DILI since Fer-1 protected primary mouse hepatocytes (PMH) in vitro from APAP [59]. This is far from conclusive as lipid peroxidation is not a prominent feature of APAP toxicity. However, iron uptake in mitochondria may be important in promoting Fenton reaction and hydroxyl radical toxicity. The role of ferroptosis in APAP-induced cell death needs to be further investigated and confirmed by in vivo experiments and using genetic knockout models.

## 3. Pathogenesis of Idiosycratic Drug-Induced Liver Injury (IDILI)

The pathogenesis of IDILI is multifactorial and complex, but several hypotheses have emerged, all of which involve the adaptive immune system. There are multiple clues that point to the involvement of the adaptive immune system in hepatotoxic reactions to drugs. For example, drugs or drug–protein adducts from IDILI compounds can activate peripheral blood lymphocytes (lymphocyte stimulation test), supporting the notion that patient specific and host immune factors contribute to IDILI [60,61,62,63].

IDILI can be further divided into two types, IDILI without immune-allergic features (the majority of cases) and IDILI with immune-allergic features (fever, eosinophilia and rash). Examples of drugs that cause immune-allergic IDILI include halothane, sulindac, dihydralazine and some anticonvulsants such as phenytoin, as well as certain antibiotics such as trimethoprim/sulfamethoxazole, cefazolin and ciprofloxacin [3]. These drugs are hypothesized to covalently bind to liver proteins such as cytochrome P450 (CYP), be presented as hapten–antigen adducts to MHC class II molecules and thus trigger an immune response that activates CD8 cytotoxic T cells to attack the liver. The T cells in turn express Fas ligand (FasL) and TNF which mediate hepatocyte cell death via death receptors abundantly expressed on the surface of hepatocytes [19]. DILI from certain drugs such as halothane, can involve cluster of differentiation 4 (CD4) T cell-regulated IgE and IgG1 antibodies to trifluoroacetyl protein adducts which may promote antibody-dependent cytotoxicity (possibly with compliment) [64]. Severe allergic skin reactions such as toxic epidermal necrosis (TEN) and Stevens–Johnson syndrome (SJS) have also been described with IDILI and carry a poor prognosis when present [64]. IDILI can mimic, induce or unmask autoimmune hepatitis (AIH), a well described autoimmune disorder of the liver involving adaptive immunity. Classical examples of drugs that cause an AIH type DILI include nitrofurantoin and minocycline, which are associated with the induction of antinuclear antibodies and the histopathological appearance of AIH on liver biopsy [62]. It is important to point out that most drugs that cause IDILI do so in the absence of classic systemic immune allergic features (fever, rash and eosinophilia); however, many are strongly HLA-linked, suggesting a liver-specific immune response. These immune responses targeted at the hepatocyte likely induce a receptor-mediated apoptotic cell death. However, non-canonical necroptotic cell death (independent of RIPK3) as suggested in idiopathic auto-immune hepatitis, could theoretically contribute [40].

### 3.1. Human Leukocyte Antigen (HLA) Associations

Genetic studies on DILI can be challenging due to the rarity of DILI occurrence, the number of drugs causing DILI, as well as the multitude of clinical manifestations. Multiple genome-wide association studies (GWAS) have identified correlations between human leukocyte antigen (HLA) polymorphisms and the occurrence of IDILI (Table 1). The implication of these studies is that IDILI is the result of the activation of an adaptive immune response. These HLA haplotype associations suggest that DILI occurs due to a genetic predisposition to an adaptive immune response as well as to the presentation and recognition of a drug-related antigen. For example, strong associations of the HLA-B*5701 polymorphic allele with flucloxacillin hepatotoxicity have been found with an 80-fold risk increase for the carriers [65,66,67]. Other examples of strong associations between HLA polymorphisms and IDILI are the increased risk of amoxicillin-clavulanate toxicity (the single most common form of IDILI) in patients with DRB1*1501 and DQB1*0602 [65]. GWAS have been performed on other drugs known to cause IDILI, such as diclofenac, ximelagatran, tacrine, tolcapone and troglitazone and risk alleles have been identified. A comprehensive list is included in Table 1 [65,66,67,68,69,70,71,72,73,74,75,76,77,78,79,80,81,82,83,84,85,86,87,88,89,90]. Although not much mechanistic evidence exists, it is thought that the presence of an HLA polymorphism or haptenization alone is not sufficient to trigger hepatic injury as most patients with these HLA polymorphisms do not develop IDILI. Stress (intra- or extra- hepatic) due to infection, inflammation or other oxidative stress burden may co-stimulate the immune response as well as elicit immune-mediated hepatic cell death. Furthermore, the issue of immune-tolerance or failure of adaptation may play a role (more below).

As more drugs are being identified and associated with DILI, and more genetic studies are being conducted with the ultimate aims of detecting, defining, studying and improving liver injury from drugs and herbal and dietary supplements (HDS), registries to carefully document DILI cases in patients have been created. The Drug-Induced Liver Injury Network (DILIN) aims to develop standardized procedures to correctly identify and characterize drug- and HDS-induced liver injury, as well as to conduct tightly controlled clinical studies on DILI [65]. Other registries such as in Spain (and Latin America), United Kingdom, Iceland and Japan also compile carefully phenotyped and adjudicated cases in hopes of better understanding the pathogenesis and genetics of IDILI and its relationship to phenotypic manifestations [91,92,93,94,95].

### 3.2. Receptor-Mediated Signaling and DILI

Classic drugs with IDILI liability may act through activation of cytokines such as fas ligand (FasL), interferon γ (IFNγ) and TNF [96]. IFNγ is a soluble cytokine secreted by immune cells, and it exerts its effects by binding to transmembrane receptors on the surface of hepatocytes and Kupffer cells [96]. The biological effects of IFNγ stimulation are the induction of the major histocompatibility complex class I and II and of nitric oxide synthase (NOS) expression, stimulation of TNF production, and the upregulation of receptors for immunoglobulins, monocytes and/or macrophages [97]. Binding of IFNγ to its receptor activates the Janus kinase (JAK) and signal transducer and activator of transcription (STAT) pathways, which increase the activity of antigen-presenting macrophages, enhance natural killer (NK) cell activation and promote leukocyte adhesion [98]. Activated Kupffer cells, in turn, produce TNF that modulates hepatocellular function, mitigates inflammatory responses and can result in apoptosis in vulnerable cells [93]. Furthermore, IFNγ and TNF can synergistically induce inducible nitric oxide synthase (Inos) expression, cause DNA fragmentation and lead to apoptosis in PMH in vitro [99,100].

There have been several in vivo experimental models of T cell-dependent apoptotic and necrotic liver injury via the activation of IFNγ described recently. Mice previously sensitized with d-Galactosamine (GalN) [101] and subsequently probed with either anti-CD3 monoclonal antibody or with the super-antigen staphylococcal enterotoxin B (SEB) developed apoptotic and necrotic liver injury and cytosolic DNA fragmentation [101,102,103]. These three models act via the activation of IFNγ and TNF, as passive immunization against TNF protects mice against liver injury induced by these agents.

Concanavalin A (Con A), a T-cell mitogenic lectin derived from the jack-bean plant, is used as an in vivo model of immune-mediated liver injury. Administration of Con A to naïve mice results in hepatitis and hepatocellular death. The exact subroutine of cell death in this model is still somewhat controversial [42,103,104,105,106]. Recently, it was reported that MLKL^−/−^ mice were resistant to cell death from Con A and this appeared to be independent of RIPK3 [40]. RIPK3 expression was absent in hepatocytes, and the direct activation of MLKL by RIPK1 was excluded which suggested the participation of an unidentified kinase that activates MLKL. Interestingly, the rapid induction of MLKL expression in this model was mediated by IFNγ signaling through STAT-1, as STAT1^−/−^ mice were also protected from Con A injury [40]. MLKL activation and translocation to the cell membrane for cell wall lysis requires phosphorylation, and only an increase in protein levels is not sufficient to induce necrosis [40]. While induction of MLKL was dependent on IFNγ/STAT-1, its activation required TNF and TNFR1^−/−^ mice were also protected from Con A hepatitis and cell death in vivo (no MLKL translocation to cell membrane) [40]. Therefore, both induction of MLKL (via IFNγ/STAT-1) and activation (p-MLKL) and translocation to the cell membrane (via TNF signaling) are necessary for MLKL induced hepatocyte death in the Con A model [40]. The exact pathway leading to RIPK1 activation in this model is unclear but presumed to be via TNF. Additionally, the direct activator of MLKL downstream of TNF is yet to be identified [40].

Activation of a hepatotoxic immune reaction using the potent NKT cell stimulator, α-galactosylceramide (α-GalCer), also results in TNF receptor-mediated apoptosis, which can be attenuated by caspase inhibitors and TNF neutralizing antibody and can be aggravated by RIPK1 knockdown in mice [107]. Liver specific RIPK1 deletion also exacerbates Con A-induced hepatitis in vivo, although in this instance TNF-mediated apoptosis is the dominant cell death subroutine [108]. Although these in vivo mouse models do no fully recapitulate human IDILI, they elucidate some molecular pathways through which one could imagine drugs exerting hepatotoxic effects leading to cell death via cell surface death receptors.

### 3.3. Hypothesis for Immune System Activation in IDILI

A few different hypotheses have been suggested to explain the idiosyncratic nature of most drug toxicities and the mode of immune activation (Table 2). The first hypothesis of IDILI is the hapten hypothesis which postulates that certain drugs are metabolized to reactive compounds that can bind to endogenous proteins and form neoantigen or “hapten” peptides that are presented to and recognized by the immune system of certain individuals with HLA polymorphisms as foreign antigens [14].

Another hypothesis termed the “pharmacological interaction” (p-i) hypothesis postulates that certain drugs can act like small molecules and directly form non-covalent interactions with MHC molecules, leading to the activation of the immune system. It is likely that the initial binding of the drug to the MHC molecule is labile, and serves as a scaffold for a T cell receptor (TCR) interaction of much higher relative affinity [109,110]. This TCR interaction is capable of generating an immunological response, as it involves T cell activation [110]. The p-i model has been described to be relevant for a series of drugs such as sulfamethoxazole, lidocaine, celecoxib, carbamazepine and ximelegatran; however, the specific sites of drug binding on the MHC–peptide complex remain unresolved for many drugs [111]. An example of the possible clinical relevance of the pi hypothesis has been proposed for the hepatotoxic drug ximelegatran since the drug does not covalently bind proteins to form neoantigens. However, in vitro studies have shown a direct inhibition by ximelegatran of peptide binding to HLA DRB1*0701, supporting direct interaction of the drug with this HLA binding site [112].

A third model, the altered peptide repertoire hypothesis, suggests that certain drugs can cause mistargeting of endogenous peptides to the wrong HLA, leading to auto-immunity. This has been described for abacavir rash and Stevens–Johnson syndrome from carbamazepine [113,114]. Abacavir binds within the F pocket of the peptide-binding groove of HLA-B*57:01, thereby changing its specificity, altering the repertoire of self-peptides presented to T cells, and thus causing the equivalent of an alloreactive T-cell response [113].

An alternative hypothesis for IDILI is the multiple determinant hypothesis, which states that multiple risk factors (such as polymorphisms, age, gender, preexisting conditions) could overlap together to induce DILI [96,115,116]. The mouse model of halothane-induced liver toxicity can be used as an example. Among the known human risk factors for halothane hepatitis are female gender, middle age, genetic predisposition, and overnight fasting [117,118]. Halothane administered to mature, female, fasted BALB/c mice caused liver injury, while fed mice were less sensitive. Male mice and immature female mice were also more resistant. Isoflurane however, which is a closely related drug and also an inhaled anesthetic that does not share the human liability for IDILI, did not cause any liver injury in these mice [118]. Therefore, unless all conditions are met and the multiple determinants are fulfilled, IDILI will not occur. This may, in part, explain why the disease is so rare in spite of the genetic polymorphisms being common in the population.

The unpredictable nature of idiosyncratic DILI may also suggest that there could be another event occurring concomitantly with drug therapy. This raises the possibility that IDILI reactions could be unmasked by inflammation occurring during drug therapy, which could interact with the action of the drug and escalate into liver injury. Such a response is oftentimes characterized by inflammatory cell infiltrates within liver lesions of patients suffering from IDILI [119,120,121,122]. These inflammagens bind to “pattern recognition receptors” such as Toll-like receptors (TLRs) on immune system cells, which initiate the activation of transcription factors and the expression of inflammatory mediators such as tumor necrosis factor α (TNFα) and IFNγ. This hypothesis of occurrences is known as the inflammatory stress hypothesis of IDILI. The inflammatory stress hypothesis has provided the first animal models in which pronounced liver injury is induced from numerous drugs associated with human IDILI, such as chlorpromazine, halothane, ranitidine, diclofenac, sulindac, and amiodarone [96,121,122]. However, the injury in this circumstance is very acute and does not have the latency features associated with human DILI from these drugs.

In experimental models of IDILI, it has been suggested that either CD4^+^ dependent antibody-mediated cytotoxicity [64], or CD8^+^ T cells-mediated cytotoxicity [123,124] result in hepatocyte death via death receptor-initiated mechanisms [62]. However, the susceptible HLA polymorphism resulting in immune activation does not fully explain hepatotoxicity on its own as most identified HLA haplotypes associated with toxicity are quite common in the general population and IDILI is a rare event. Thus, not all individuals with the susceptible HLA polymorphism exhibit IDILI when exposed to the drug, which may be due to the liver’s inherent state of immune privilege and modulation of immune-tolerance [62].

### 3.4. Immune-Tolerance and Adaptation

In order to avoid inflammatory reaction due to its routine exposure to foreign antigens, the liver is in a constant state of immune tolerance. Antigens are ingested and continuously introduced to the liver where they are metabolized and either excreted via the biliary system or metabolized [62]. The liver’s immune tolerance has been the focus of scientific study since the 1960s, when experimental transplantation studies revealed that allogeneic liver grafts can be established and maintained in animal models without immunosuppression [124,125]. HLA antigen matching is not necessary for successful liver transplants, and liver transplantation is the only solid organ transplant in which complete weaning of immunosuppression can be achieved in up to 20% of cases [126]. This dampening of the liver’s immune response is multifaceted and implemented by the various non-parenchymal cells residing in the liver. This may be the main reason why many patients exposed to potentially hepatotoxic drugs have transient liver enzyme abnormalities that normalize with continuous exposure (clinical adaptation) [106]. A classic example of this was described in 1975. Patients exposed to the anti-tuberculosis drug isoniazid (INH) in an inpatient psychiatry unit were followed with serial blood tests and 38% of patients on INH experienced abnormally elevated liver enzymes (some with hyperbilirubinemia) which subsided in the majority of patients despite continued treatment with INH [127]. More recently, several studies and animal models have shown that when these intrinsic liver auto-immunity checkpoints were experimentally bypassed, drugs that normally would not result in liver injury or only cause transient DILI, caused T cell activation with persistent and more severe DILI [64,122,123,128].

The parenchymal epithelial cells, hepatocytes, along with the cholangiocytes which line the bile ducts, are the functional units of the liver. However, mammalian liver contains various other cell types, referred to as non-parenchymal cells (NPC), which are essential for normal biologic and immunologic functions. These include liver sinusoidal endothelial cells (LSECs, which constitute the wall of the liver sinusoids), Kupffer cells (KCs) which are resident liver macrophages, stellate cells (HSCs) which are pericytes found in perisinusoidal space, liver-associated lymphocytes and dendritic cells. LSECs produce cytokines, activate CD4^+^ T cells and function as a barrier between leukocytes or other macromolecules present in the sinusoidal lumen and hepatocytes [129,130,131]. KCs, which are specialized machrophages, are located mainly in the periportal sinusoids so they can phagocytose and eliminate antigens and pathogens entering the liver parenchyme via venous blood from the intestines [125]. The liver’s immune tolerance is dependent on the autocrine and paracrine effects of cytokines secreted by KCs as well as on LPS stimulation of immune cells and antigen presenting cells (KC and LSECs particularly) [132]. KCs and LSECs express cytokines such as interleukin 10 (IL-10), TGFβ, TNFα and prostaglandins either constitutively or in response to LPS, resulting in downregulation of leukocyte adhesion to LSECs, expansion of regulatory T cells (T-regs) and abrogation of T cell activation, all of which lead to an increased immune tolerance in the liver [106,133,134,135,136,137].

Elegant studies have been conducted to demonstrate that bypassing the liver’s intrinsic immunity checkpoints leads to the development of IDILI. Programmed cell death protein 1 (PD1) and cytotoxic T-lymphocyte-associated protein 4 (CTLA4) are important immune checkpoint mediators which are involved in inducing hepatic immune tolerance [138]. PD1^−/−^ mice treated with anti-CTLA4 antibodies prior to treatment with amodiaquine (AQ) suffered from greater liver injury than from AQ alone [123]. PD1 null mice treated with anti-CTLA4 antibody have impaired immune-adaptive responses and constitute a valid animal model of AQ IDILI, characterized by significant liver dysfunction and injury [128]. In another study, Chakraborty and colleagues provide evidence that immune tolerance in the liver can be overcome in the murine model of halothane-induced allergic hepatitis [64]. In this model, female BALB/c mice received two doses of halothane to become sensitized to the drug. Each dose elicited ALT elevations, perivenous necrosis and inflitration of CD11b+Gr-1^high^ cells in the liver 24 h post drug administration followed by complete normalization of ALT (clinical adaptation). A subpopulation of myeloid-derived suppressor cells within the CD11b+Gr-1^high^ cell fraction was also observed, which inhibited the proliferation of both CD4^+^ and CD8^+^ T cells, and can induce immune tolerance. When these CD11b+Gr-1^high^ immunosuppressor cells were depleted from the liver with Gr-1 antibody treatment (24 h before halothane), reappearance of enhanced liver injury was observed nine days after halothane rechallenge [64]. The mice treated with Gr-1 antibody also displayed delayed severe inflammation, necrosis, increased eosinophil infiltration and T cell response [64]. Interestingly, the liver injury in the AQ model was CD8 cytotoxic T cell-mediated apoptosis whereas in the halothane model, the injury was CD4 T cell-induced humoral (antibody and complement) mediated and lytic necrosis [64,122].

## 4. Direct Hepatocyte Toxicity

### Acetaminophen Toxicity

Acetaminophen (APAP) is the quintessential hepatotoxin. APAP causes DILI in a predictable, dose-dependent and intrinsically hepatotoxic manner and is the most common single cause of DILI in the United States and the UK [139]. Acetaminophen-related adverse events continue to be a public health burden [140]. APAP overdose is the leading cause for calls to Poison Control Centers (>100,000/year) and accounts for more than 50,000 emergency room visits, 2600 hospitalizations, and nearly 500 deaths per year as a result of APAP-associated acute liver failure [139,140,141,142].

APAP is absorbed rapidly in healthy adults and is extensively metabolized mainly by the liver, although there may be some metabolism of the drug in the gut and kidney. Only 2–5% of the ingested dose of APAP is excreted unchanged in the urine [143,144,145]. The major metabolites of APAP are the glucuronide and sulfate conjugates, while a minor fraction is converted in the liver by CYP2E1 to a highly reactive toxic electrophilic arylating metabolite, *N*-acetyl-*p*-benzoquinoneimine (NAPQI). NAPQI is normally quickly inactivated by being preferentially conjugated with reduced GSH in the liver, and then is excreted in the bile and urine as cysteine and mercapturic acid conjugates. When large doses of APAP are ingested, glucuronidation and sulfation pathways are saturated and the remaining APAP is shunted to the CYP system. Due to overwhelming NAPQI production, GSH becomes depleted and NAPQI remains in the liver where it can covalently bind to cell protein thiols, presumably altering their functions and eventually leading to acute hepatic necrosis [146]. In vitro, in the presence of reduced GSH in isolated rat hepatocytes, NAPQI can either be reduced back to APAP or covalently linked to GSH to form a conjugate [147,148]. In addition to covalent binding, NAPQI can oxidize protein thiol groups, leading to the formation of inter-protein crosslinking, disulfide bridges or mixed disulfides. Furthermore, NAPQI decreases are equivalents of NADPH with concomitant reduction of molecular oxygen thus generating ROS, which in turn can initiate lipid peroxidation (LPO), though this is not a prominent feature [149,150]. Though NAPQI is produced in the ER, it is sufficiently stable to pass into mitochondria or be exported and attack LSECs, potentially contributing to the hemorrhagic nature of hepatic injury [16]. Micro RNAs (miR) which are small non-coding RNAs that regulate gene expression have recently been the focus of studies in various liver diseases [151]. miRs can affect drug metabolism by regulating the expression of the CYPs and drug transporters and serve as biomarkers for liver injury during DILI [151,152]. Recently, in vitro studies using stem cell-derived hepatocytes and primary human hepatocytes have demonstrated a role for miR-324 in the regulation of phase II enzymes glutathione-S-transferase (GST1) and sulfotransferase 2A1 (SULT2A1) [153]. Pre-treatment with microRNA-324 antagomir resulted in increased SULT2A1, higher glutathione levels and protection against APAP in vitro by interfering with APAP metabolism [153].

NAPQI and consequent mitochondrial-derived ROS damage mitochondrial DNA and activate the c-Jun N terminal Kinase (JNK) signaling pathway leading to amplification of mitochondrial ROS, which eventually leads to the opening of the mitochondrial membrane permeability transition pore (MPT). MPT opening results in the collapse of mitochondrial membrane potential, and thus the cessation of ATP synthesis as well as to the release of intermembrane proteins which trigger necrotic cell death [154]. The mode of cell death in APAP hepatotoxicity has long been viewed as oncotic necrosis [155,156] and not apoptosis, as there is no caspase activation after APAP overdose [157] in spite of mitochondrial rupture and release of intermembrane proteins [158]. In addition, caspase inhibitors have been shown to be ineffective in protecting the liver against APAP toxicity [159].

ROS activate upstream kinases such as glycogen synthase kinase 3 β (GSK3β), RIPK1, Protein kinase C α (PKCα), higher order mitogen activated protein kinases (MAPKs) such as Mixed-lineage kinase 3 (MLK3), apoptosis signal regulating kinase 1 (ASK1) and mitogen-activated protein kinase kinase 4 (MKK4), ultimately leading to JNK activation. MLK3 is activated by oxidative stress, and results in the first phase of JNK activation [160], while ASK1 regulates the late phase of APAP-induced JNK activation [161]. Activated JNK (p-JNK) then binds to its target Sab on the mitochondrial outer membrane, phosphorylating it and leading to a positive feedback loop of ROS production and JNK activation ultimately leading to MPT [8]. Interfering with this pathway at any point, by knockdown, knockout or inhibition of the proteins protects hepatocytes from cell death [8,160,161,162,163,164]. Importantly, knockout of SH3 binding protein 5 (Sab) or inhibition of JNK binding to Sab prevents APAP toxicity as well, indicating that the interaction of these two proteins is a critical step in mitochondrial collapse and cell death [163,164,165]. JNK phosphorylates Sab on the cytoplasmic side of the mitochondria, leading to the release of a protein tyrosine phosphatase type 6 (PTPN6) from Sab in the intermembrane space [166]. After release from Sab, PTPN6 is activated and transfers to the inner membrane, where it dephosphorylates and inactivates Src in the intermembrane space. Src dephosphorylation requires a platform protein, docking protein 4 (DOK4), on the inner membrane. Active Src is required to maintain electron transport in the inner membrane [166]. When Src is inactivated, the electron transport chain (ETC) chain is blocked and ROS production is enhanced. Knockdown of mitochondrial DOK4 or PTPN6 inhibited the inactivation of mitochondrial p-Src and the effect of p-JNK on mitochondria, indicating that these proteins are crucial for APAP mitochondria toxicity via JNK [166].

NAPQI covalent binding in the ER may also induce the unfolded protein response (UPR) and ER stress [167] response, which may contribute to APAP hepatotoxicity as a site for ASK1/JNK activation.

The penultimate event before mitochondrial collapse is MPT and inhibition of pore opening by the pharmacological inhibition of cyclophilin D, with cyclosporin A providing protection against MTP opening and APAP-induced necrosis [168,169]. Similarly, cyclophilin D-deficient mice were protected against low dose APAP toxicity [170], but not against a higher dose [171].

## 5. Preclinical Models for Screening Drugs for IDILI

A variety of screening approaches for DILI liability beyond the standard in vivo mouse, rat and monkey treatment have been studied. Use of cell lines (HepG2, HepRG) and primary human hepatocytes (PHH) in sandwich culture, micropatterned co-culture and spheroids with tight cell interactions is commonly used to study drug toxicity. There are, however, some pitfalls with the in vitro models. A major drawback of cell lines is that they are derived from a single donor with uniform genetics, thus rendering the study of a spectrum of polymorphisms impossible [172]. In vitro exposure of mouse liver mitochondria has been used as a preclinical test for a drug’s potential for IDILI [173]. Using isolated mouse liver mitochondria, Porceddu and colleagues developed a high throughput method for screening mitochondrial toxicity of a large set of DILI and non-DILI drugs using readouts such as cytochrome c release, membrane permeabilization (swelling), collapse of membrane potential and alterations in respiration driven by succinate or malate/glutamate [173]. Toxicity to mitochondria in vitro had a sensitivity of 92–94% and high positive predictive value of 82–89% for a hepatotoxic outcome [173]. PHH are likely the most accurate representation of human liver but even these cells when cultured for more than 72 h lose their differentiation in vitro. The addition of sandwich culture and matrices such as Matrigel can delay the decline of hepatocyte function. However, even with the overlay cultures, hepatocyte function decreases to a mere 10% after a few days [174]. While PHH remain the gold standard for in vitro DILI experiments, in order to recapitulate genetic diversity among humans and understand inter-individual differences in susceptibility to DILI, induced pluripotent stem cells (iPSCs) have been emerging as a new tool to study drug toxicity [174]. Like the other models, iPSCs have limitations as well, mainly due to poor differentiation of cells to hepatocytes [175]. The readouts for these in vitro models include oxidative stress, mitochondrial dysfunction, and cell viability. Despite using high drug concentrations and short-term exposures, these assays perform well in predicting a drug’s liability for inducing IDILI. Considering that IDILI usually has a latency of weeks to months, the question is why do these acute models perform well and do they reveal the relevant mechanism in human IDILI. One possibility is that these models are a surrogate for the fact that most DILI drugs are lipophilic and undergo extensive metabolism. Alternatively, it is possible that these assays inform on the stress-inducing effects of the drugs which may be a prerequisite for the development of an adaptive immune response in genetically susceptible individuals carrying the relevant HLA polymorphism. This can be viewed as a danger signal to enhance immune activation. Another possibility is that the stress inducing phenomenon elicited by the drug or metabolites may sensitize hepatocytes to the lethal effects of an adaptive immune response. At present, these are unresolved issues. Specialized animal models have also been explored though not widely adopted in the screening of drugs. Examples include: concomitant administration of LPS with drugs, the use of immune checkpoint inhibitors, and humanized mice [62,174]. All of these approaches suffer from the inability to capture the variability of the human population and the unique susceptibility of a subgroup to hepatotoxicity (e.g., HLA polymorphism).

## 6. Clinical Aspects

Most of the hypotheses and data discussed here pertain to in vitro and in vivo experimental models. Therefore, with the exception of HLA polymorphism studies performed on IDILI patients, drawing direct conclusions about human IDILI in relation to cell death pathways is speculative. Therefore, mechanistic steps should be viewed critically in light of the fact that recapitulation of human IDILI has largely not been experimentally feasible. However, these mechanistic studies in animal models and in vitro systems are important in developing therapeutic solutions and can also lead to the discovery of sorely needed biomarkers for IDILI and cell death subroutines [176].

Another major challenge in the field is causality assessment since DILI is a diagnosis of exclusion. Clinical databases and studies use various methods of causality assessment and there is a lack of unanimity among experts as to which method is optimum [65,177,178,179,180]. Biomarker discovery holds promise in this arena in providing objective tests to diagnose DILI. Most clinicians diagnose DILI based on previous knowledge of a drug, temporal relationship and by ruling out other causes of liver injury which is applicable to patient safety [180]. However, more rigorous approaches are required in publications and specialized contexts.

## 7. Conclusions

**Figure 2:**
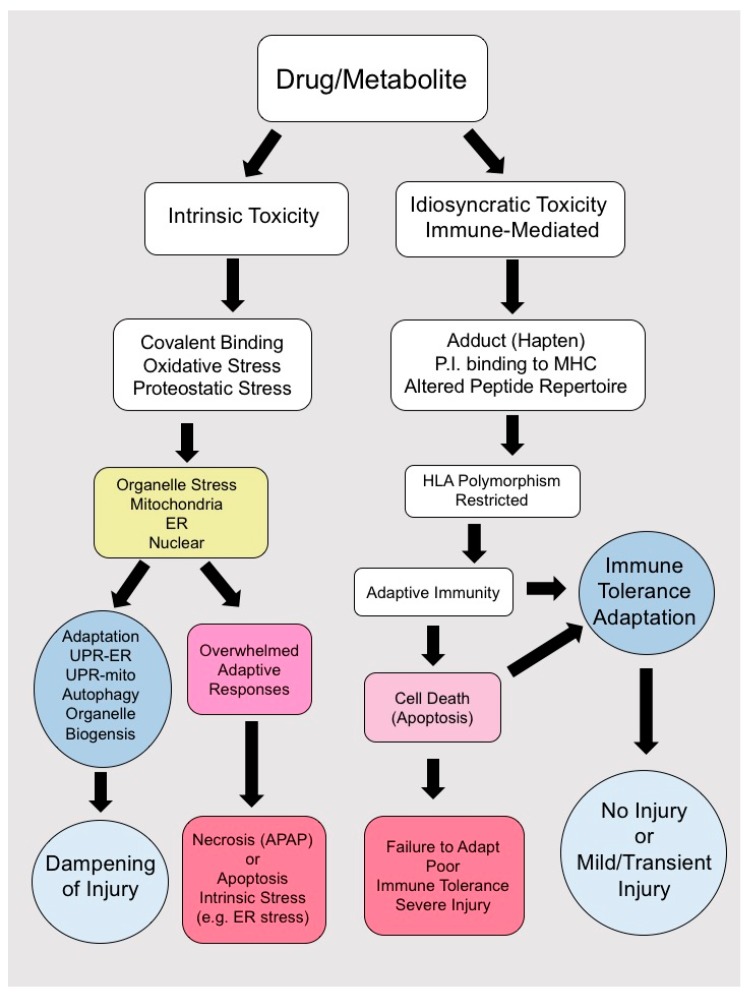
Drugs or toxic metabolites can cause intrinsic predictable toxicity by binding to intracellular proteins, generating reactive oxygen species (ROS) and inducing organelle stress. If the stress is minor, organelle adaptive responses (such as the unfolded protein responses in the ER or mitochondria) will compensate and adapt and the injury will be dampened. If these responses are overwhelmed, cell death follows. Most drugs cause an idiosyncratic and immune-mediated injury which is HLA-associated and only occurs in individuals with the susceptible HLA polymorphism. Due to the process of adaptation and the liver’s inherent capacity for immune tolerance, most individuals exposed to a toxic drug even in the presence of a susceptible HLA polymorphism will experience mild and transient injury or no injury at all. In a minority of individuals, failure of adaptation results in persistent and severe injury.

The cascade of events leading to DILI depends largely on the type of hepatotoxin (Table 1). Human IDILI by definition has been difficult to recapitulate and study in animal models, as many patient-specific factors contribute to drug toxicity [63,172,181,182,183]. Exposure of hepatocytes to parent drug or reactive metabolites may induce a variety of patient specific intracellular stress responses and adaptive mechanisms, which illicit danger signals activating the innate and adaptive immune system [172,173] (Figure 2). Most patients exposed to IDILI drugs and their metabolites develop transient mild abnormalities, which resolve with continued exposure (clinical adaptation). Certain individuals with polymorphisms in HLA molecules (Table 1) when exposed to these highly immunogenic compounds may develop overt clinical IDILI, although a multitude of conditions need to be met for IDILI to occur and not all patients with the susceptible HLA haplotypes develop IDILI. Thus, IDILI is the result of a complex interplay between potentially immunogenic drugs and drug metabolites and the host’s immune response and capacity for immune tolerance (Figure 2).

The molecular pathway of APAP toxicity is the subject of much investigation. The APAP metabolite, NAPQI, is a mitochondrial toxin. Mitochondrial toxicity and the generation of ROS results in the activation of signaling molecules such as RIPK1, GSK3β, PKCα, MLK3, ASK1 and JNK [4,14]. This cascade of signaling events ultimately results in the phosphorylation of JNK and its translocation to mitochondria where it binds to Sab and results in the release of a protein phosphatase that inactivates intermitochondria Src [166]. More investigation is needed to identify how Src interacts with the ETC to influence mitochondrial function.

Cell death is a dominant feature of DILI. There are two main types of cell death, apoptosis and necrosis (Figure 1), and both can be implicated and involved in DILI. It is not clear if other modes of cell death (such as pyroptosis and ferroptosis) contribute to DILI, while autophagy (mitophagy) is mainly protective. Most drugs that cause idiosyncratic toxicity or IDILI do so through the participation of the adaptive immune system and HLA polymorphisms. Although no human data exists, one can speculate that in these instances, DILI and the resultant immune-mediated cell death is likely executed via death receptors and is apoptotic. However, the possibility of necroptosis (either canonical or non-canonical) in human DILI has not been studied or excluded. Understanding the mechanisms of subroutines of hepatocellular death in DILI will be critical in directing therapy to mitigate the cell death and avert acute liver failure. Potential approaches include singly or in combination antagonizing death receptors, caspases, specific kinases such as ASK1, JNK, RIPK1, RIPK3. However, there is theoretical danger in inhibiting one cell death pathway as this may activate another as a default mechanism. For example, in certain contexts and cell types, inhibition of apoptotic caspases may activate necroptosis, or inhibition of necroptotic RIPK1 or RIPK3 may activate apoptosis. Therefore, targeting multiple pathways may be the best approach.

## Figures and Tables

**Table 1:** Associations between human leukocyte antigen (HLA) and idiosyncratic drug-induced liver injury (IDILI).

Drug	Reference	Human Leukocyte Antigen Associations
Abacavir	[70]	B*57:01
Allopurinol	[81]	B*58:01 ^
Amoxicillin-Clavulanate	[71,72,73]	A*02:01, B*18:01, DRB1*1501, DQB1*0602, DRB1*07 ^‡^, A*3002, DQB1*0402
Anti-Tuberculous Drugs	[74,75,76]	DQB1*02:01, DQB1*05:02, DQA1*01:02 ^‡^, B*57 ^#^, DRB1*03
Antiretroviral Drugs	[76]	B*57 ^#^
Carbamazepine	[82]	B*15:11 ^, A*31:01 ^
Clometacin	[84]	B*08
Diclofenac	[85]	DRB1*13
Fenofibrate	[79]	A*33:01
Flucloxacillin	[66,67,86]	B*57:01, DRB1*07:01, DQB1*03:03, DRB1*15 ^‡^
Flupirtine	[83]	DRB1*16:01, DQB1*05:02
Lapatinib	[77]	DQA1*02:01, DQB1*02:02, DRB1*07:01
Lumiracoxib	[87]	DRB1*15:01, DQB1*06:02, DRB5*01:01, DQA1*01:02
Minocyclin	[78]	HLA B*35:02, B*35:02
Nevirapine	[80]	DRB1*01:02, DRB1*01, B*58:01
Pazopanib	[88]	B*57:01
Phenobarbital	[82]	B*51:01 ^
Terbinafine	[80]	A*33:01
Ticlopidine	[89]	A*33:03, A*33:01, B*44:03, Cw*1403, DRB1*1302, DQB1*0604
Tiopronine	[90]	A*33 B44 DR ^
Ximelagatran	[72]	DRB1*0701, DQA1*0201
Zonisamide	[82]	A*02:07 ^

^‡^ Haplotype decreases risk of toxicity; ^ Haplotype reported in Japanese patients; ^#^ Haplotype reported in Ethiopian patients.

**Table 2:** Hypotheses of immune system activation.

Name of Hypothesis	Definition
Hapten hypothesis	Reactive metabolites are generated from drugs that can bind to endogenous proteins and form neoantigens, activating the immune system.
Pharmacological Interaction (p-i) Hypothesis	Certain drugs can act like small molecules and directly form non-covalent interactions with MHC molecules altering their binding pocket.
The altered peptide repertoire hypothesis	Drugs induce mistargeting of endogenous peptides to the wrong HLA leading to autoimmunity.
Multiple determinant hypothesis	Multiple risk factors (i.e., polymorphisms, age, gender), are necessary and overlap together to induce DILI.
Inflammatory stress hypothesis	A small inflammation occurring during drug therapy could interact with the action of the drug and escalate into liver injury.

## Abbreviations

α-GalCer	α-Galactosylceramide
AIH	Autoimmune hepatitis
ALF	Acute liver failure
APAF-1	Apoptotic peptidase activating factor-1
APAP	Acetaminophen
AQ	Amodiaquine
ASK1	Apoptosis signal regulating kinase 1
ATG	Autophagy
ATP	Adenosine triphosphate
Bax	Bcl-2-like protein 4
Bcl-2	B-cell lymphoma 2
c-FLIP	FLICE inhibitory protein
CD4	Cluster of differentiation 4
CD8	Cluster of differentiation 8
CD95	Cluster of differentiation 95, also known as the FAS receptor
cIAP1/2	Cellular inhibitor of apoptosis 1/2
Con A	Concanavalin A
CTLA4	Cytotoxic T-lymphocyte-associated protein 4
CYP	Cytochrome P450
DILI	Drug-induced liver injury
DILIN	Drug-Induced Liver Injury Network
DNA	Deoxyribonucleic acid
DOK4	Docking protein 4
DR	Death receptors
ER	Endoplasmic reticulum
ETC	Electron transport chain
FADD	Fas-associated protein with death domain
FasL	Fas ligand
Fer-1	Ferrostatins
FLICE	Fas-associating protein with death domain-like interleukin-1 β-converting enzyme
GalN	D-galactosamine
GPX4	Glutathione peroxidase 4
GSDMD	Gasdermin D
GSH	Glutathione
GSK3β	Glycogen synthase kinase 3 β
GST1	Glutathione-S-transferase
GWAS	Genome-wide association studies
HDS	Herbal and dietary supplements
HLA	Human leukocyte antigen
HSC	Hepatic stellate cells
IDILI	Idiosycratic drug-induced liver injury
IFNγ	Interferon γ
IL-10	Interleukin 10
INH	Isoniazide
iNOS	Inducible nitric oxide synthase
iPSC	Induced pluripotent stem cell
JAK	Janus kinase
JNK	c-Jun terminal kinase
KC	Kupffer cells
LPO	Lipid peroxidation
LPS	Lipopolysaccharide
LSECs	Liver sinusoidal endothelial cells
MAPK	Mitogen-activated protein kinases
MHC	Major histocompatibility complex
MiR	micro RNA
MKK4	Mitogen-activated protein kinase kinase 4
MLK3	Mixed-lineage kinase 3
MLKL	Mixed lineage domain like
MOMP	Mitochondrial outer membrane permeabilization
MPT	Membrane permeability transition
NAPQI	*N*-acetyl-*p*-benzoquinoneimine
Nec	Necrostatins
NFκB	Nuclear factor kappa-light-chain-enhancer of activated B cells
NK	Natural killer cells
NKT	Natural killer T cells
NOS	Nitric oxide synthase
NPC	Non-parenchymal cells
PD1	Programmed cell death protein 1
PKCα	Protein kinase C α
PHH	Primary human hepatocytes
PMH	Primary mouse hepatocytes
PTPN6	Protein tyrosine phosphatase type 6
RHIM	RIP homology interaction motif
RIPK1/RIP1	Receptor interacting serine/threonine kinase 1
RIPK3/RIP3	Receptor interacting serine/threonine kinase 3
ROS	Reactive oxygen species
Sab	SH3 binding protein 5
SEB	Super-antigen staphylococcal enterotoxin B
SJS	Stevens–Johnson syndrome
STAT	Signal transducer and activator of transcription
STAT1	Signal transducer and activator of transcription 1
SULT2A1	sulfotransferase 2A1
T-regs	Regulatory T cells
TAB2/3	TAK1-binding protein 2/3
TAK1	Transforming growth factor-β-activated kinase 1
tBid	Cleaved Bid
TCR	T cell receptor
TEN	Toxic epidermal necrosis
TGFβ	Transforming growth factor β
TNF	Tumor necrosis factor
TNFR1	Tumor necrosis factor receptor 1
TNFα	Tumor necrosis factor α
TRADD	TNFR-associated death domain
TRAF2/5	TNFR-associated factor 2/ 5
TRAIL-R1/R2	TNF-related apoptosis-inducing ligand receptor 1/2
UPR	Unfolded protein response
WT	Wild type

## References

[1] Motamedi N.D.L., Kaplowitz N., McQueen C. (2017). Clinical considerations of drug-induced hepatotoxicity. Comprehensive Toxicology.

[2] Kaplowitz N. (2005). Idiosyncratic drug hepatotoxicity. Nat. Rev. Drug Discov..

[3] Yuan L., Kaplowitz N. (2013). Mechanisms of drug-induced liver injury. Clin. Liver Dis..

[4] Dara L., Han D., Kaplowitz N. (2012). Mechanisms of Cell Death and Relevance to Drug Toxicity.

[5] Larson A.M., Polson J., Fontana R.J., Davern T.J., Lalani E., Hynan L.S., Reisch J.S., Schiodt F.V., Ostapowicz G., Shakil A.O. (2005). Acetaminophen-induced acute liver failure: Results of a united states multicenter, prospective study. Hepatology.

[6] Nathwani R.A., Kaplowitz N. (2006). Drug hepatotoxicity. Clin. Liver Dis..

[7] Senior J.R. (2007). Drug hepatotoxicity from a regulatory perspective. Clin. Liver Dis..

[8] Kaplowitz N., Win S., Than T.A., Liu Z.X., Dara L. (2015). Targeting signal transduction pathways which regulate necrosis in acetaminophen hepatotoxicity. J. Hepatol..

[9] Lucena M.I., Andrade R.J., Kaplowitz N., Garcia-Cortes M., Fernandez M.C., Romero-Gomez M., Bruguera M., Hallal H., Robles-Diaz M., Rodriguez-Gonzalez J.F. (2009). Phenotypic characterization of idiosyncratic drug-induced liver injury: The influence of age and sex. Hepatology.

[10] Denk H. (2002). Drug-induced liver injury. Verh. Dtsch. Ges. Pathol..

[11] Chen M., Borlak J., Tong W. (2013). High lipophilicity and high daily dose of oral medications are associated with significant risk for drug-induced liver injury. Hepatology.

[12] Kaplowitz N. (2013). Avoiding idiosyncratic DILI: Two is better than one. Hepatology.

[13] Bell L.N., Chalasani N. (2009). Epidemiology of idiosyncratic drug-induced liver injury. Semin. Liver Dis..

[14] Dara L.L.Z., Kaplowitz N., Ding W.X., Yin X.M. (2017). Cell death in drug induced liver injury. Cell Death in Liver Disease.

[15] Odin J.A., Huebert R.C., Casciola-Rosen L., LaRusso N.F., Rosen A. (2001). Bcl-2-dependent oxidation of pyruvate dehydrogenase-e2, a primary biliary cirrhosis autoantigen, during apoptosis. J. Clin. Investig..

[16] DeLeve L.D., Wang X., Kaplowitz N., Shulman H.M., Bart J.A., van der Hoek A. (1997). Sinusoidal endothelial cells as a target for acetaminophen toxicity: Direct action versus requirement for hepatocyte activation in different mouse strains. Biochem. Pharmacol..

[17] DeLeve L.D., Wang X., Kuhlenkamp J.F., Kaplowitz N. (1996). Toxicity of azathioprine and monocrotaline in murine sinusoidal endothelial cells and hepatocytes: The role of glutathione and relevance to hepatic venoocclusive disease. Hepatology.

[18] Galluzzi L., Maiuri M.C., Vitale I., Zischka H., Castedo M., Zitvogel L., Kroemer G. (2007). Cell death modalities: Classification and pathophysiological implications. Cell Death Differ..

[19] Guicciardi M.E., Gores G.J. (2009). Life and death by death receptors. FASEB J..

[20] Tian Z., Chen Y., Gao B. (2013). Natural killer cells in liver disease. Hepatology.

[21] Protzer U., Maini M.K., Knolle P.A. (2012). Living in the liver: Hepatic infections. Nat. Rev. Immunol..

[22] Micheau O., Tschopp J. (2003). Induction of TNF receptor I-mediated apoptosis via two sequential signaling complexes. Cell.

[23] Ashkenazi A., Salvesen G. (2014). Regulated cell death: Signaling and mechanisms. Annu. Rev. Cell Dev. Biol..

[24] Karin M., Lin A. (2002). NFκB at the crossroads of life and death. Nat. Immunol..

[25] Micheau O., Lens S., Gaide O., Alevizopoulos K., Tschopp J. (2001). NFκB signals induce the expression of c-flip. Mol. Cell. Biol..

[26] Nagai H., Matsumaru K., Feng G., Kaplowitz N. (2002). Reduced glutathione depletion causes necrosis and sensitization to tumor necrosis factor-α-induced apoptosis in cultured mouse hepatocytes. Hepatology.

[27] Pierce R.H., Campbell J.S., Stephenson A.B., Franklin C.C., Chaisson M., Poot M., Kavanagh T.J., Rabinovitch P.S., Fausto N. (2000). Disruption of redox homeostasis in tumor necrosis factor-induced apoptosis in a murine hepatocyte cell line. Am. J. Pathol..

[28] Lou H., Kaplowitz N. (2007). Glutathione depletion down-regulates tumor necrosis factor α-induced NFκB activity via IκB kinase-dependent and -independent mechanisms. J. Biol. Chem..

[29] Han D., Hanawa N., Saberi B., Kaplowitz N. (2006). Hydrogen peroxide and redox modulation sensitize primary mouse hepatocytes to TNF-induced apoptosis. Free Radic. Biol. Med..

[30] Kim H.E., Du F., Fang M., Wang X. (2005). Formation of apoptosome is initiated by cytochrome *c*-induced dATP hydrolysis and subsequent nucleotide exchange on APAF-1. Proc. Natl. Acad. Sci. USA.

[31] Zou H., Henzel W.J., Liu X., Lutschg A., Wang X. (1997). APAF-1, a human protein homologous to *C. elegans* CED-4, participates in cytochrome c-dependent activation of caspase-3. Cell.

[32] Acehan D., Jiang X., Morgan D.G., Heuser J.E., Wang X., Akey C.W. (2002). Three-dimensional structure of the apoptosome: Implications for assembly, procaspase-9 binding, and activation. Mol. Cell.

[33] Bratton S.B., Salvesen G.S. (2010). Regulation of the APAF-1-caspase-9 apoptosome. J. Cell Sci..

[34] Newton K., Manning G. (2016). Necroptosis and inflammation. Annu. Rev. Biochem..

[35] Degterev A., Hitomi J., Germscheid M., Ch’en I.L., Korkina O., Teng X., Abbott D., Cuny G.D., Yuan C., Wagner G. (2008). Identification of RIP1 kinase as a specific cellular target of necrostatins. Nat. Chem. Biol..

[36] Murphy J.M., Czabotar P.E., Hildebrand J.M., Lucet I.S., Zhang J.G., Alvarez-Diaz S., Lewis R., Lalaoui N., Metcalf D., Webb A.I. (2013). The pseudokinase MLKL mediates necroptosis via a molecular switch mechanism. Immunity.

[37] Sun L., Wang H., Wang Z., He S., Chen S., Liao D., Wang L., Yan J., Liu W., Lei X. (2012). Mixed lineage kinase domain-like protein mediates necrosis signaling downstream of RIP3 kinase. Cell.

[38] Wang H., Sun L., Su L., Rizo J., Liu L., Wang L.F., Wang F.S., Wang X. (2014). Mixed lineage kinase domain-like protein MLKL causes necrotic membrane disruption upon phosphorylation by RIP3. Mol. Cell.

[39] Degterev A., Huang Z., Boyce M., Li Y., Jagtap P., Mizushima N., Cuny G.D., Mitchison T.J., Moskowitz M.A., Yuan J. (2005). Chemical inhibitor of nonapoptotic cell death with therapeutic potential for ischemic brain injury. Nat. Chem. Biol..

[40] Gunther C., He G.W., Kremer A.E., Murphy J.M., Petrie E.J., Amann K., Vandenabeele P., Linkermann A., Poremba C., Schleicher U. (2016). The pseudokinase MLKL mediates programmed hepatocellular necrosis independently of RIPK3 during hepatitis. J. Clin. Investig..

[41] Afonso M.B., Rodrigues P.M., Carvalho T., Caridade M., Borralho P., Cortez-Pinto H., Castro R.E., Rodrigues C.M. (2015). Necroptosis is a key pathogenic event in human and experimental murine models of non-alcoholic steatohepatitis. Clin. Sci..

[42] Dara L., Liu Z.X., Kaplowitz N. (2016). Questions and controversies: The role of necroptosis in liver disease. Cell Death Discov..

[43] Newton K., Dugger D.L., Wickliffe K.E., Kapoor N., de Almagro M.C., Vucic D., Komuves L., Ferrando R.E., French D.M., Webster J. (2014). Activity of protein kinase RIPK3 determines whether cells die by necroptosis or apoptosis. Science.

[44] Degterev A., Zhou W., Maki J.L., Yuan J. (2014). Assays for necroptosis and activity of RIP kinases. Methods Enzymol..

[45] Sun X., Lee J., Navas T., Baldwin D.T., Stewart T.A., Dixit V.M. (1999). RIP3, a novel apoptosis-inducing kinase. J. Biol. Chem..

[46] Kasof G.M., Prosser J.C., Liu D., Lorenzi M.V., Gomes B.C. (2000). The RIP-like kinase, RIP3, induces apoptosis and NFκB nuclear translocation and localizes to mitochondria. FEBS Lett..

[47] Dara L., Johnson H., Suda J., Win S., Gaarde W., Han D., Kaplowitz N. (2015). Receptor interacting protein kinase 1 mediates murine acetaminophen toxicity independent of the necrosome and not through necroptosis. Hepatology.

[48] An J., Mehrhof F., Harms C., Lattig-Tunnemann G., Lee S.L., Endres M., Li M., Sellge G., Mandic A.D., Trautwein C. (2013). ARC is a novel therapeutic approach against acetaminophen-induced hepatocellular necrosis. J. Hepatol..

[49] Ramachandran A., McGill M.R., Xie Y., Ni H.M., Ding W.X., Jaeschke H. (2013). Receptor interacting protein kinase 3 is a critical early mediator of acetaminophen-induced hepatocyte necrosis in mice. Hepatology.

[50] Deutsch M., Graffeo C.S., Rokosh R., Pansari M., Ochi A., Levie E.M., Van Heerden E., Tippens D.M., Greco S., Barilla R. (2015). Divergent effects of RIP1 or RIP3 blockade in murine models of acute liver injury. Cell Death Dis..

[51] Galluzzi L., Vitale I., Abrams J.M., Alnemri E.S., Baehrecke E.H., Blagosklonny M.V., Dawson T.M., Dawson V.L., El-Deiry W.S., Fulda S. (2012). Molecular definitions of cell death subroutines: Recommendations of the nomenclature committee on cell death 2012. Cell Death Differ..

[52] Ni H.M., Bockus A., Boggess N., Jaeschke H., Ding W.X. (2012). Activation of autophagy protects against acetaminophen-induced hepatotoxicity. Hepatology.

[53] Aglietti R.A., Dueber E.C. (2017). Recent insights into the molecular mechanisms underlying pyroptosis and gasdermin family functions. Trends Immunol..

[54] Kayagaki N., Stowe I.B., Lee B.L., O’Rourke K., Anderson K., Warming S., Cuellar T., Haley B., Roose-Girma M., Phung Q.T. (2015). Caspase-11 cleaves gasdermin D for non-canonical inflammasome signalling. Nature.

[55] Shi J., Zhao Y., Wang K., Shi X., Wang Y., Huang H., Zhuang Y., Cai T., Wang F., Shao F. (2015). Cleavage of GSDMD by inflammatory caspases determines pyroptotic cell death. Nature.

[56] Cao J.Y., Dixon S.J. (2016). Mechanisms of ferroptosis. Cell. Mol. Life Sci..

[57] Friedmann Angeli J.P., Schneider M., Proneth B., Tyurina Y.Y., Tyurin V.A., Hammond V.J., Herbach N., Aichler M., Walch A., Eggenhofer E. (2014). Inactivation of the ferroptosis regulator GPX4 triggers acute renal failure in mice. Nat. Cell Biol..

[58] Dixon S.J., Lemberg K.M., Lamprecht M.R., Skouta R., Zaitsev E.M., Gleason C.E., Patel D.N., Bauer A.J., Cantley A.M., Yang W.S. (2012). Ferroptosis: An iron-dependent form of nonapoptotic cell death. Cell.

[59] Lorincz T., Jemnitz K., Kardon T., Mandl J., Szarka A. (2015). Ferroptosis is involved in acetaminophen induced cell death. Pathol. Oncol. Res..

[60] Monshi M.M., Faulkner L., Gibson A., Jenkins R.E., Farrell J., Earnshaw C.J., Alfirevic A., Cederbrant K., Daly A.K., French N. (2013). Human leukocyte antigen (HLA)-B*57:01-restricted activation of drug-specific T cells provides the immunological basis for flucloxacillin-induced liver injury. Hepatology.

[61] Tsutsui H., Terano Y., Sakagami C., Hasegawa I., Mizoguchi Y., Morisawa S. (1992). Drug-specific T cells derived from patients with drug-induced allergic hepatitis. J. Immunol..

[62] Dara L., Liu Z.X., Kaplowitz N. (2015). Mechanisms of adaptation and progression in idiosyncratic drug induced liver injury, clinical implications. Liver Int..

[63] Lauschke V.M., Ingelman-Sundberg M. (2016). The importance of patient-specific factors for hepatic drug response and toxicity. Int. J. Mol. Sci..

[64] Chakraborty M., Fullerton A.M., Semple K., Chea L.S., Proctor W.R., Bourdi M., Kleiner D.E., Zeng X., Ryan P.M., Dagur P.K. (2015). Drug-induced allergic hepatitis developed in mice when myeloid-derived suppressor cells were depleted prior to halothane treatment. Hepatology.

[65] Chalasani N., Bonkovsky H.L., Fontana R., Lee W., Stolz A., Talwalkar J., Reddy K.R., Watkins P.B., Navarro V., Barnhart H. (2015). Features and outcomes of 899 patients with drug-induced liver injury: The dilin prospective study. Gastroenterology.

[66] Daly A.K., Donaldson P.T., Bhatnagar P., Shen Y., Pe’er I., Floratos A., Daly M.J., Goldstein D.B., John S., Nelson M.R. (2009). HLA-B*5701 genotype is a major determinant of drug-induced liver injury due to flucloxacillin. Nat. Genet..

[67] Wuillemin N., Adam J., Fontana S., Krahenbuhl S., Pichler W.J., Yerly D. (2013). HLA haplotype determines hapten or p-i t cell reactivity to flucloxacillin. J. Immunol..

[68] Bhogaraju A., Nazeer S., Al-Baghdadi Y., Rahman M., Wrestler F., Patel N. (1999). Diclofenac-associated hepatitis. South. Med. J..

[69] Daly A.K., Aithal G.P., Leathart J.B., Swainsbury R.A., Dang T.S., Day C.P. (2007). Genetic susceptibility to diclofenac-induced hepatotoxicity: Contribution of UGT2B7, CYP2C8, and ABCC2 genotypes. Gastroenterology.

[70] Mallal S., Phillips E., Carosi G., Molina J.M., Workman C., Tomazic J., Jagel-Guedes E., Rugina S., Kozyrev O., Cid J.F. (2008). *HLA-B*5701* screening for hypersensitivity to abacavir. N. Engl. J. Med..

[71] Hautekeete M.L., Horsmans Y., van Waeyenberge C., Demanet C., Henrion J., Verbist L., Brenard R., Sempoux C., Michielsen P.P., Yap P.S. (1999). HLA association of amoxicillin-clavulanate--induced hepatitis. Gastroenterology.

[72] Kindmark A., Jawaid A., Harbron C.G., Barratt B.J., Bengtsson O.F., Andersson T.B., Carlsson S., Cederbrant K.E., Gibson N.J., Armstrong M. (2008). Genome-wide pharmacogenetic investigation of a hepatic adverse event without clinical signs of immunopathology suggests an underlying immune pathogenesis. Pharmacogenomics J..

[73] Lucena M.I., Molokhia M., Shen Y., Urban T.J., Aithal G.P., Andrade R.J., Day C.P., Ruiz-Cabello F., Donaldson P.T., Stephens C. (2011). Susceptibility to amoxicillin-clavulanate-induced liver injury is influenced by multiple HLA class I and II alleles. Gastroenterology.

[74] Sharma S.K., Balamurugan A., Saha P.K., Pandey R.M., Mehra N.K. (2002). Evaluation of clinical and immunogenetic risk factors for the development of hepatotoxicity during antituberculosis treatment. Am. J. Respir. Crit. Care Med..

[75] Chen R., Zhang Y., Tang S., Lv X., Wu S., Sun F., Xia Y., Zhan S.Y. (2015). The association between *HLA-DQB1* polymorphism and antituberculosis drug-induced liver injury: A case-control study. J. Clin. Pharm. Ther..

[76] Petros Z., Kishikawa J., Makonnen E., Yimer G., Habtewold A., Aklillu E. (2017). *HLA-B*57* allele is associated with concomitant anti-tuberculosis and antiretroviral drugs induced liver toxicity in ethiopians. Front. Pharmacol..

[77] Spraggs C.F., Budde L.R., Briley L.P., Bing N., Cox C.J., King K.S., Whittaker J.C., Mooser V.E., Preston A.J., Stein S.H. (2011). *HLA-DQA1*02:01* is a major risk factor for lapatinib-induced hepatotoxicity in women with advanced breast cancer. J. Clin. Oncol..

[78] Urban T.J., Nicoletti P., Chalasani N., Serrano J., Stolz A., Daly A., Aithal G., Dillon J., Navarro V., Odin J. (2017). Minocycline hepatotoxicity: Clinical characterization and identification of *HLA-B*35:02* as a risk factor. J. Hepatol..

[79] Nicoletti P., Aithal G.P., Bjornsson E.S., Andrade R.J., Sawle A., Arrese M., Barnhart H.X., Bondon-Guitton E., Hayashi P.H., Bessone F. (2017). Association of liver injury from specific drugs, or groups of drugs, with polymorphisms in HLA and other genes in a genome-wide association study. Gastroenterology.

[80] Phillips E., Bartlett J.A., Sanne I., Lederman M.M., Hinkle J., Rousseau F., Dunn D., Pavlos R., James I., Mallal S.A. (2013). Associations between HLA-DRB1*0102, HLA-B*5801, and hepatotoxicity during initiation of nevirapine-containing regimens in south africa. J. Acquir. Immune Defic. Syndr..

[81] Uetrecht J. (2008). Idiosyncratic drug reactions: Past, present, and future. Chem. Res. Toxicol..

[82] Saito Y., Kodama S., Sugiyama E., Nakamura R. (2015). Predictive genomic markers for severe adverse drug reactions. Yakugaku Zasshi.

[83] Nicoletti P., Werk A.N., Sawle A., Shen Y., Urban T.J., Coulthard S.A., Bjornsson E.S., Cascorbi I., Floratos A., Stammschulte T. (2016). *HLA-DRB1*16:01-DQB1*05:02* is a novel genetic risk factor for flupirtine-induced liver injury. Pharmacogenet. Genom..

[84] Berson A., Freneaux E., Larrey D., Lepage V., Douay C., Mallet C., Fromenty B., Benhamou J.P., Pessayre D. (1994). Possible role of HLA in hepatotoxicity: An exploratory study in 71 patients with drug-induced idiosyncratic hepatitis. J. Hepatol..

[85] Daly A.K., Day C.P. (2009). Genetic association studies in drug-induced liver injury. Semin. Liver Dis..

[86] Russmann S., Kaye J.A., Jick S.S., Jick H. (2005). Risk of cholestatic liver disease associated with flucloxacillin and flucloxacillin prescribing habits in the UK: Cohort study using data from the UK general practice research database. Br. J. Clin. Pharmacol..

[87] Singer J.B., Lewitzky S., Leroy E., Yang F., Zhao X., Klickstein L., Wright T.M., Meyer J., Paulding C.A. (2010). A genome-wide study identifies HLA alleles associated with lumiracoxib-related liver injury. Nat. Genet..

[88] Xu C.F., Johnson T., Wang X., Carpenter C., Graves A., Warren L., Xue Z., King K.S., Fraser D.J., Stinnett S. (2016). *HLA-B*57:01* confers susceptibility to pazopanib-associated liver injury in patients with cancer. Clin. Cancer Res..

[89] Hirata K., Takagi H., Yamamoto M., Matsumoto T., Nishiya T., Mori K., Shimizu S., Masumoto H., Okutani Y. (2008). Ticlopidine-induced hepatotoxicity is associated with specific human leukocyte antigen genomic subtypes in japanese patients: A preliminary case-control study. Pharmacogenom. J..

[90] Kurosaki M., Takagi H., Mori M. (2000). HLA-A33/B44/DR6 is highly related to intrahepatic cholestasis induced by tiopronin. Dig. Dis. Sci..

[91] Andrade R.J., Lucena M.I., Fernandez M.C., Pelaez G., Pachkoria K., Garcia-Ruiz E., Garcia-Munoz B., Gonzalez-Grande R., Pizarro A., Duran J.A. (2005). Drug-induced liver injury: An analysis of 461 incidences submitted to the spanish registry over a 10-year period. Gastroenterology.

[92] Bjornsson E.S. (2015). Drug-induced liver injury: An overview over the most critical compounds. Arch. Toxicol..

[93] Bessone F., Hernandez N., Lucena M.I., Andrade R.J., Latin DILI Network, Spanish DILI Registry (2016). The Latin American DILI registry experience: A successful ongoing collaborative strategic initiative. Int. J. Mol. Sci..

[94] Bjornsson E.S., Bergmann O.M., Bjornsson H.K., Kvaran R.B., Olafsson S. (2013). Incidence, presentation, and outcomes in patients with drug-induced liver injury in the general population of iceland. Gastroenterology.

[95] Takikawa H., Murata Y., Horiike N., Fukui H., Onji M. (2009). Drug-induced liver injury in japan: An analysis of 1676 cases between 1997 and 2006. Hepatol. Res..

[96] Roth R.A., Maiuri A.R., Ganey P.E. (2017). Idiosyncratic drug-induced liver injury: Is drug-cytokine interaction the linchpin?. J. Pharmacol. Exp. Ther..

[97] Kusters S., Gantner F., Kunstle G., Tiegs G. (1996). Interferon γ plays a critical role in T cell-dependent liver injury in mice initiated by concanavalin A. Gastroenterology.

[98] Schroder K., Hertzog P.J., Ravasi T., Hume D.A. (2004). Interferon-γ: An overview of signals, mechanisms and functions. J. Leukoc. Biol..

[99] Morita M., Watanabe Y., Akaike T. (1995). Protective effect of hepatocyte growth factor on interferon-γ-induced cytotoxicity in mouse hepatocytes. Hepatology.

[100] Vodovotz Y., Kim P.K., Bagci E.Z., Ermentrout G.B., Chow C.C., Bahar I., Billiar T.R. (2004). Inflammatory modulation of hepatocyte apoptosis by nitric oxide: In vivo, in vitro, and in silico studies. Curr. Mol. Med..

[101] Nagaki M., Muto Y., Ohnishi H., Yasuda S., Sano K., Naito T., Maeda T., Yamada T., Moriwaki H. (1994). Hepatic injury and lethal shock in galactosamine-sensitized mice induced by the superantigen staphylococcal enterotoxin B. Gastroenterology.

[102] Gantner F., Leist M., Jilg S., Germann P.G., Freudenberg M.A., Tiegs G. (1995). Tumor necrosis factor-induced hepatic DNA fragmentation as an early marker of T cell-dependent liver injury in mice. Gastroenterology.

[103] Gantner F., Leist M., Lohse A.W., Germann P.G., Tiegs G. (1995). Concanavalin A-induced T-cell-mediated hepatic injury in mice: The role of tumor necrosis factor. Hepatology.

[104] Tiegs G., Hentschel J., Wendel A. (1992). A T cell-dependent experimental liver injury in mice inducible by concanavalin a. J. Clin. Investig..

[105] Leist M., Gantner F., Naumann H., Bluethmann H., Vogt K., Brigelius-Flohe R., Nicotera P., Volk H.D., Wendel A. (1997). Tumor necrosis factor-induced apoptosis during the poisoning of mice with hepatotoxins. Gastroenterology.

[106] Dara L., Liu Z., Kaplowitz N. (2016). A murder mystery in the liver: Who done it and how?. J. Clin. Investig..

[107] Suda J., Dara L., Yang L., Aghajan M., Song Y., Kaplowitz N., Liu Z.X. (2016). Knockdown of RIPK1 markedly exacerbates murine immune-mediated liver injury through massive apoptosis of hepatocytes, independent of necroptosis and inhibition of nf-kappab. J. Immunol..

[108] Filliol A., Piquet-Pellorce C., Le Seyec J., Farooq M., Genet V., Lucas-Clerc C., Bertin J., Gough P.J., Dimanche-Boitrel M.T., Vandenabeele P. (2016). RIPK1 protects from TNF-α-mediated liver damage during hepatitis. Cell Death Dis..

[109] Von Greyerz S., Bultemann G., Schnyder K., Burkhart C., Lotti B., Hari Y., Pichler W.J. (2001). Degeneracy and additional alloreactivity of drug-specific human αβ^+^ T cell clones. Int. Immunol..

[110] Von Greyerz S., Zanni M.P., Frutig K., Schnyder B., Burkhart C., Pichler W.J. (1999). Interaction of sulfonamide derivatives with the TCR of sulfamethoxazole-specific human αβ^+^ T cell clones. J. Immunol..

[111] Pichler W.J. (2002). Pharmacological interaction of drugs with antigen-specific immune receptors: The p-i concept. Curr. Opin. Allergy Clin. Immunol..

[112] Grove J.I., Aithal G.P. (2015). Human leukocyte antigen genetic risk factors of drug-induced liver toxicology. Expert Opin. Drug Metab. Toxicol..

[113] Ostrov D.A., Grant B.J., Pompeu Y.A., Sidney J., Harndahl M., Southwood S., Oseroff C., Lu S., Jakoncic J., de Oliveira C.A. (2012). Drug hypersensitivity caused by alteration of the MHC-presented self-peptide repertoire. Proc. Natl. Acad. Sci. USA.

[114] Wei C.Y., Chung W.H., Huang H.W., Chen Y.T., Hung S.I. (2012). Direct interaction between HLA-B and carbamazepine activates T cells in patients with stevens-johnson syndrome. J. Allergy Clin. Immunol..

[115] Li A.P. (2002). A review of the common properties of drugs with idiosyncratic hepatotoxicity and the “multiple determinant hypothesis” for the manifestation of idiosyncratic drug toxicity. Chem. Biol. Interact..

[116] Ulrich R.G. (2007). Idiosyncratic toxicity: A convergence of risk factors. Annu. Rev. Med..

[117] Ray D.C., Drummond G.B. (1991). Halothane hepatitis. Br. J. Anaesth..

[118] Dugan C.M., MacDonald A.E., Roth R.A., Ganey P.E. (2010). A mouse model of severe halothane hepatitis based on human risk factors. J. Pharmacol. Exp. Ther..

[119] Khouri M.R., Saul S.H., Dlugosz A.A., Soloway R.D. (1987). Hepatocanalicular injury associated with vitamin a derivative etretinate. An idiosyncratic hypersensitivity reaction. Dig. Dis. Sci..

[120] Fukano M., Amano S., Sato J., Yamamoto K., Adachi H., Okabe H., Fujiyama Y., Bamba T. (2000). Subacute hepatic failure associated with a new antidiabetic agent, troglitazone: A case report with autopsy examination. Hum. Pathol..

[121] Deng X., Luyendyk J.P., Ganey P.E., Roth R.A. (2009). Inflammatory stress and idiosyncratic hepatotoxicity: Hints from animal models. Pharmacol. Rev..

[122] Shaw P.J., Ganey P.E., Roth R.A. (2010). Idiosyncratic drug-induced liver injury and the role of inflammatory stress with an emphasis on an animal model of trovafloxacin hepatotoxicity. Toxicol. Sci..

[123] Mak A., Uetrecht J. (2015). The role of CD8 T cells in amodiaquine-induced liver injury in PD1^−/−^ mice cotreated with anti-CTLA-4. Chem. Res. Toxicol..

[124] Metushi I.G., Hayes M.A., Uetrecht J. (2015). Treatment of PD-1^−/−^ mice with amodiaquine and anti-CTLA4 leads to liver injury similar to idiosyncratic liver injury in patients. Hepatology.

[125] Calne R.Y., Sells R.A., Pena J.R., Davis D.R., Millard P.R., Herbertson B.M., Binns R.M., Davies D.A. (1969). Induction of immunological tolerance by porcine liver allografts. Nature.

[126] Knolle P.A., Gerken G. (2000). Local control of the immune response in the liver. Immunol. Rev..

[127] Adams D.H., Sanchez-Fueyo A., Samuel D. (2015). From immunosuppression to tolerance. J. Hepatol..

[128] Mitchell J.R., Long M.W., Thorgeirsson U.P., Jollow D.J. (1975). Acetylation rates and monthly liver function tests during one year of isoniazid preventive therapy. Chest.

[129] Uetrecht J., Kaplowitz N. (2015). Inhibition of immune tolerance unmasks drug-induced allergic hepatitis. Hepatology.

[130] Wisse E., de Zanger R.B., Charels K., van der Smissen P., McCuskey R.S. (1985). The liver sieve: Considerations concerning the structure and function of endothelial fenestrae, the sinusoidal wall and the space of disse. Hepatology.

[131] Fraser R., Dobbs B.R., Rogers G.W. (1995). Lipoproteins and the liver sieve: The role of the fenestrated sinusoidal endothelium in lipoprotein metabolism, atherosclerosis, and cirrhosis. Hepatology.

[132] Lohse A.W., Knolle P.A., Bilo K., Uhrig A., Waldmann C., Ibe M., Schmitt E., Gerken G., Meyer Zum Buschenfelde K.H. (1996). Antigen-presenting function and B7 expression of murine sinusoidal endothelial cells and kupffer cells. Gastroenterology.

[133] Knolle P.A., Germann T., Treichel U., Uhrig A., Schmitt E., Hegenbarth S., Lohse A.W., Gerken G. (1999). Endotoxin down-regulates T cell activation by antigen-presenting liver sinusoidal endothelial cells. J. Immunol..

[134] Bissell D.M., Wang S.S., Jarnagin W.R., Roll F.J. (1995). Cell-specific expression of transforming growth factor-β in rat liver. Evidence for autocrine regulation of hepatocyte proliferation. J. Clin. Investig..

[135] Knolle P.A., Uhrig A., Protzer U., Trippler M., Duchmann R., Meyer zum Buschenfelde K.H., Gerken G. (1998). Interleukin-10 expression is autoregulated at the transcriptional level in human and murine kupffer cells. Hepatology.

[136] Knolle P., Schlaak J., Uhrig A., Kempf P., zum Buschenfelde K.H.M., Gerken G. (1995). Human Kupffer cells secrete IL-10 in response to lipopolysaccharide (LPS) challenge. J. Hepatol..

[137] Rieder H., Ramadori G., Allmann K.H., zum Buschenfelde K.H.M. (1990). Prostanoid release of cultured liver sinusoidal endothelial cells in response to endotoxin and tumor necrosis factor: Comparison with umbilical vein endothelial cells. J. Hepatol..

[138] Dieter P., Schulze-Specking A., Karck U., Decker K. (1987). Prostaglandin release but not superoxide production by rat Kupffer cells stimulated in vitro depends on Na^+^/H^+^ exchange. Eur. J. Biochem..

[139] Mak A., Johnston A., Uetrecht J. (2017). Effects of immunization and checkpoint inhibition on amodiaquine-induced liver injury. J. Immunotoxicol..

[140] Lee W.M. (2004). Acetaminophen and the U.S. acute liver failure study group: Lowering the risks of hepatic failure. Hepatology.

[141] Major J.M., Zhou E.H., Wong H.L., Trinidad J.P., Pham T.M., Mehta H., Ding Y., Staffa J.A., Iyasu S., Wang C. (2016). Trends in rates of acetaminophen-related adverse events in the United States. Pharmacoepidemiol. Drug Saf..

[142] Nourjah P., Ahmad S.R., Karwoski C., Willy M. (2006). Estimates of acetaminophen (paracetomal)-associated overdoses in the United States. Pharmacoepidemiol. Drug Saf..

[143] Rowden A.K., Norvell J., Eldridge D.L., Kirk M.A. (2005). Updates on acetaminophen toxicity. Med. Clin. N. Am..

[144] Prescott L.F. (1974). Gastrointestinal absorption of drugs. Med. Clin. N. Am..

[145] Josting D., Winne D., Bock K.W. (1976). Glucuronidation of paracetamol, morphine and 1-naphthol in the rat intestinal loop. Biochem. Pharmacol..

[146] Mitchell J.R., Jollow D.J., Potter W.Z., Davis D.C., Gillette J.R., Brodie B.B. (1973). Acetaminophen-induced hepatic necrosis. I. Role of drug metabolism. J. Pharmacol. Exp. Ther..

[147] Mitchell J.R., Thorgeirsson S.S., Potter W.Z., Jollow D.J., Keiser H. (1974). Acetaminophen-induced hepatic injury: Protective role of glutathione in man and rationale for therapy. Clin. Pharmacol. Ther..

[148] Van de Straat R., de Vries J., Kulkens T., Debets A.J., Vermeulen N.P. (1986). Paracetamol, 3-monoalkyl- and 3,5-dialkyl derivatives: Comparison of their microsomal cytochrome P-450 dependent oxidation and toxicity in freshly isolated hepatocytes. Biochem. Pharmacol..

[149] Moldeus P. (1978). Paracetamol metabolism and toxicity in isolated hepatocytes from rat and mouse. Biochem. Pharmacol..

[150] Gibson J.D., Pumford N.R., Samokyszyn V.M., Hinson J.A. (1996). Mechanism of acetaminophen-induced hepatotoxicity: Covalent binding versus oxidative stress. Chem. Res. Toxicol..

[151] Pumford N.R., Hinson J.A., Benson R.W., Roberts D.W. (1990). Immunoblot analysis of protein containing 3-(cystein-S-yl) acetaminophen adducts in serum and subcellular liver fractions from acetaminophen-treated mice. Toxicol. Appl. Pharmacol..

[152] Lauschke V.M., Mkrtchian S., Ingelman-Sundberg M. (2017). The role of micrornas in liver injury at the crossroad between hepatic cell death and regeneration. Biochem. Biophys. Res. Commun..

[153] Wang K., Zhang S., Marzolf B., Troisch P., Brightman A., Hu Z., Hood L.E., Galas D.J. (2009). Circulating micrornas, potential biomarkers for drug-induced liver injury. Proc. Natl. Acad. Sci. USA.

[154] Szkolnicka D., Lucendo-Villarin B., Moore J.K., Simpson K.J., Forbes S.J., Hay D.C. (2016). Reducing hepatocyte injury and necrosis in response to paracetamol using noncoding RNAs. Stem Cells Transl. Med..

[155] Jaeschke H. (2011). Reactive oxygen and mechanisms of inflammatory liver injury: Present concepts. J. Gastroenterol. Hepatol..

[156] Gujral J.S., Knight T.R., Farhood A., Bajt M.L., Jaeschke H. (2002). Mode of cell death after acetaminophen overdose in mice: Apoptosis or oncotic necrosis?. Toxicol. Sci..

[157] Jaeschke H., Gujral J.S., Bajt M.L. (2004). Apoptosis and necrosis in liver disease. Liver Int..

[158] Lawson J.A., Fisher M.A., Simmons C.A., Farhood A., Jaeschke H. (1999). Inhibition of Fas receptor (CD95)-induced hepatic caspase activation and apoptosis by acetaminophen in mice. Toxicol. Appl. Pharmacol..

[159] Bajt M.L., Cover C., Lemasters J.J., Jaeschke H. (2006). Nuclear translocation of endonuclease G and apoptosis-inducing factor during acetaminophen-induced liver cell injury. Toxicol. Sci..

[160] Jaeschke H., Williams C.D., Farhood A. (2011). No evidence for caspase-dependent apoptosis in acetaminophen hepatotoxicity. Hepatology.

[161] Sharma M., Gadang V., Jaeschke A. (2012). Critical role for mixed-lineage kinase 3 in acetaminophen-induced hepatotoxicity. Mol. Pharmacol..

[162] Nakagawa H., Maeda S., Hikiba Y., Ohmae T., Shibata W., Yanai A., Sakamoto K., Ogura K., Noguchi T., Karin M. (2008). Deletion of apoptosis signal-regulating kinase 1 attenuates acetaminophen-induced liver injury by inhibiting c-Jun N-terminal kinase activation. Gastroenterology.

[163] Gunawan B.K., Liu Z.X., Han D., Hanawa N., Gaarde W.A., Kaplowitz N. (2006). c-Jun N-terminal kinase plays a major role in murine acetaminophen hepatotoxicity. Gastroenterology.

[164] Hanawa N., Shinohara M., Saberi B., Gaarde W.A., Han D., Kaplowitz N. (2008). Role of JNK translocation to mitochondria leading to inhibition of mitochondria bioenergetics in acetaminophen-induced liver injury. J. Biol. Chem..

[165] Win S., Than T.A., Han D., Petrovic L.M., Kaplowitz N. (2011). C-jun n-terminal kinase (JNK)-dependent acute liver injury from acetaminophen or tumor necrosis factor (TNF) requires mitochondrial sab protein expression in mice. J. Biol. Chem..

[166] Huo Y., Win S., Than T.A., Yin S., Ye M., Hu H., Kaplowitz N. (2017). Antcin h protects against acute liver injury through disruption of the interaction of c-Jun N-terminal kinase with mitochondria. Antioxid. Redox Signal..

[167] Win S., Than T.A., Min R.W., Aghajan M., Kaplowitz N. (2016). c-Jun N-terminal kinase mediates mouse liver injury through a novel Sab (SH3BP5)-dependent pathway leading to inactivation of intramitochondrial Src. Hepatology.

[168] Uzi D., Barda L., Scaiewicz V., Mills M., Mueller T., Gonzalez-Rodriguez A., Valverde A.M., Iwawaki T., Nahmias Y., Xavier R. (2013). Chop is a critical regulator of acetaminophen-induced hepatotoxicity. J. Hepatol..

[169] Kon K., Kim J.S., Jaeschke H., Lemasters J.J. (2004). Mitochondrial permeability transition in acetaminophen-induced necrosis and apoptosis of cultured mouse hepatocytes. Hepatology.

[170] Masubuchi Y., Suda C., Horie T. (2005). Involvement of mitochondrial permeability transition in acetaminophen-induced liver injury in mice. J. Hepatol..

[171] Ramachandran A., Lebofsky M., Baines C.P., Lemasters J.J., Jaeschke H. (2011). Cyclophilin D deficiency protects against acetaminophen-induced oxidant stress and liver injury. Free Radic. Res..

[172] Loguidice A., Boelsterli U.A. (2011). Acetaminophen overdose-induced liver injury in mice is mediated by peroxynitrite independently of the cyclophilin d-regulated permeability transition. Hepatology.

[173] Lin C., Khetani S.R. (2016). Advances in engineered liver models for investigating drug-induced liver injury. BioMed Res. Int..

[174] Porceddu M., Buron N., Roussel C., Labbe G., Fromenty B., Borgne-Sanchez A. (2012). Prediction of liver injury induced by chemicals in human with a multiparametric assay on isolated mouse liver mitochondria. Toxicol. Sci..

[175] Khetani S.R., Bhatia S.N. (2008). Microscale culture of human liver cells for drug development. Nat. Biotechnol..

[176] Gerbal-Chaloin S., Funakoshi N., Caillaud A., Gondeau C., Champon B., Si-Tayeb K. (2014). Human induced pluripotent stem cells in hepatology: Beyond the proof of concept. Am. J. Pathol..

[177] Teschke R., Schulze J., Eickhoff A., Danan G. (2017). Drug induced liver injury: Can biomarkers assist RUCAM in causality assessment?. Int. J. Mol. Sci..

[178] Fontana R.J., Watkins P.B., Bonkovsky H.L., Chalasani N., Davern T., Serrano J., Rochon J., Group D.S. (2009). Drug-induced liver injury network (DILIN) prospective study: Rationale, design and conduct. Drug Saf..

[179] Lucena M.I., Camargo R., Andrade R.J., Perez-Sanchez C.J., de la Cuesta F.S. (2001). Comparison of two clinical scales for causality assessment in hepatotoxicity. Hepatology.

[180] Danan G., Teschke R. (2016). Rucam in drug and herb induced liver injury: The update. Int. J. Mol. Sci..

[181] Kaplowitz N. (2001). Causality assessment versus guilt-by-association in drug hepatotoxicity. Hepatology.

[182] Chen M., Suzuki A., Borlak J., Andrade R.J., Lucena M.I. (2015). Drug-induced liver injury: Interactions between drug properties and host factors. J. Hepatol..

[183] Russmann S., Kullak-Ublick G.A., Grattagliano I. (2009). Current concepts of mechanisms in drug-induced hepatotoxicity. Curr. Med. Chem..

